# TOP mRNPs: Molecular Mechanisms and Principles of Regulation

**DOI:** 10.3390/biom10070969

**Published:** 2020-06-27

**Authors:** Eric Cockman, Paul Anderson, Pavel Ivanov

**Affiliations:** 1 Brigham and Women’s Hospital, Harvard Medical School, Boston, MA 02115, USA; ecockman@bwh.harvard.edu; 2 Brigham and Women’s Hospital, Harvard Medical School, Harvard Initiative for RNA Medicine, Boston, MA 02115, USA; panderson@bwh.harvard.edu

**Keywords:** 5’ Terminal Oligopyrimidine, RNA binding proteins, LARP1, translation regulation, RNA

## Abstract

The cellular response to changes in the surrounding environment and to stress requires the coregulation of gene networks aiming to conserve energy and resources. This is often achieved by downregulating protein synthesis. The 5’ Terminal OligoPyrimidine (5’ TOP) motif-containing mRNAs, which encode proteins that are essential for protein synthesis, are the primary targets of translational control under stress. The TOP motif is a cis-regulatory RNA element that begins directly after the m7G cap structure and contains the hallmark invariant 5’-cytidine followed by an uninterrupted tract of 4–15 pyrimidines. Regulation of translation via the TOP motif coordinates global protein synthesis with simultaneous co-expression of the protein components required for ribosome biogenesis. In this review, we discuss architecture of TOP mRNA-containing ribonucleoprotein complexes, the principles of their assembly, and the modes of regulation of TOP mRNA translation.

## 1. Translation Initiation and Regulation

**Figure 1:**
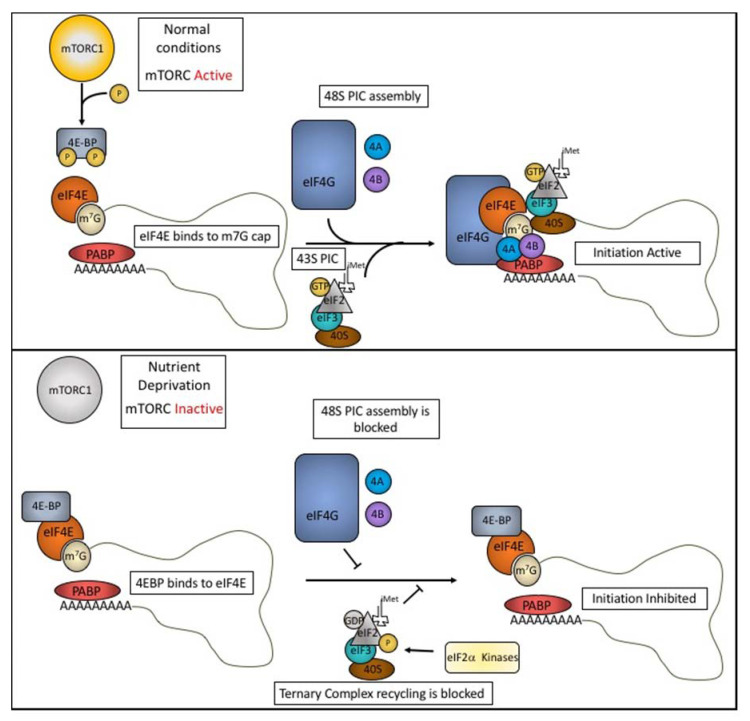
Cap-dependent translation initiation and regulation. Upper panel: Under normal conditions, mTORC1 phosphorylates and inactivates 4E-BPs. eIF4E binds to the m7G cap of the transcript and recruits eIF4G, eIF4A (4A), and eIF4B (4B) forming the eIF4F cap binding complex. eIF4F recruits the 43S pre-initiation complex (PIC) consisting of eIF3, the ternary complex (eIF2, GTP, and the initiator Methionine tRNA), and the 40S small ribosomal subunit to form the 48S PIC. Lower Panel: During nutrient deprivation, mTORC1 is inactivated resulting in hypo-phosphorylated 4E-BP. 4E-BP can then bind to eIF4E and block assembly of eIF4F, in turn, halting translation initiation. The eIF2α kinases are activated during stress responses and phosphorylate eIF2α. This phosphorylation interferes with GDP exchange and renders the ternary complex inactive.

The 5’ 7-methylguanosine (m7G) cap found at the 5’ terminus of most mRNAs plays a major role in the initiation of translation. Its recruitment of eIF4E, a major cap binding protein, is the first step in the assembly of a pre-initiation complex (PIC) that includes eIF4G, eIF4A, eIF3, the eIF2α-GTP-tRNAiMet ternary complex, and the 40S small ribosomal subunit. This 43S PIC scans the 5’ untranslated region until the tRNAiMet anticodon pairs with the AUG start codon (Figure 1, upper panel). This recognition event triggers GTP hydrolysis, release of several initiation factors, and recruitment of the 60S ribosomal subunit to allow translation to begin. The availability of active eIF2α-GTP-tRNAiMet ternary complex is influenced by environmental conditions. Under optimal growth conditions, the GTP-charged “active” ternary complex is abundant and mRNA translation is unimpaired. Under stress conditions (e.g., oxidative stress, viral infections, amino acid starvation, and ER stress), phosphorylation of eIF2α by one of several stress-activated kinases (e.g., heme-regulated inhibitor kinase (HR1), protein kinase RNA (PKR), general control non-repressible-2 (GCN2), and PKR-like ER Kinase (PERK) [1,2,3,4]) inhibits the GDP:GTP exchange reaction, depleting eIF2α-GTP-tRNAiMet, and inhibiting translation initiation [5]. For a more thorough review of translational initiation and how it is regulated, see [6,7].

The mammalian target of rapamycin complex 1 (mTORC1) plays an important role in reprogramming protein synthesis in cells subjected to metabolic stress. This is accomplished by altering the structure of the translation initiation complex to modulate the efficiency of protein synthesis. Under growth conditions, active mTORC1 phosphorylates the eIF4E Binding Proteins (4E-BPs), to prevent their interaction with eIF4E (Figure 1, upper panel). This allows eIF4E to complex with eIF4G and eIF4A to assemble initiation complexes that support protein synthesis. Under starvation conditions, inactivation of mTORC1 results in a lack of phosphorylation of 4E-BP. Unphosphorylated 4E-BP binds to eIF4E, displacing eIF4G, and inhibiting protein synthesis [8]. Thus, phosphorylation of eIF2α and dephosphorylation of 4E-BP play important roles in reprogramming protein synthesis during stress.

## 2. The 5’ TOP Motif

Protein synthesis is an essential function that requires an abundant investment of both energy and resources from cells. A myriad of ribosomal proteins, initiation, and elongation factors are necessary for translation to support cell growth and proliferation. Under normal conditions, ribosomes and translation factors are synthesized in coordination with cellular demands. However, under adverse conditions, such as amino acid deprivation or hypoxia, translation is halted, and the energy and resources required to support protein synthesis are redirected to resolve stress-induced cellular damage. mRNAs encoding proteins require for translation are distinguished by a 5’ Terminal OligoPyrimidine (5’ TOP) motif. The 5’ TOP motif begins with a m^7^G capped C nucleotide followed by a run of approximately 4-15 pyrimidines [9] often followed by a G-rich region [10,11] (Table A1). The 5’ TOP motif is highly conserved and is found in all 79 human ribosomal proteins as well as non-ribosomal proteins involved in translation including multiple subunits of eIF3, eIF4A, eEF2, and poly(A) binding protein (PABP) [12]. This shared TOP motif allows cells to quickly modulate the expression of proteins involved in ribosome production and protein synthesis in response to changes in cellular homeostasis. 

## 3. Regulation of TOP mRNAs

The expression of proteins encoded by TOP mRNAs is sensitive to the cellular energy state. When amino acid or oxygen levels are depleted, translation of TOP mRNAs is decreased [13,14]. Translation of TOP mRNAs can be stimulated by replenishment of growth factors and nutrients through the closely integrated mTORC1 and PI3K pathways. Several studies have demonstrated the importance of the mTORC1 signaling pathway on the regulation of TOP mRNAs. Disruption of mTORC1 signaling using inhibitors, such as rapamycin and Torin1 [15], leads to a marked decrease in translation of transcripts containing the 5’ TOP motif [16]. By employing ribosome profiling techniques, Thoreen et al. were able to study the translational rate of RNA transcripts in mouse cells treated with Torin1 [17]. This study aimed to identify the classes of transcripts that are regulated by mTORC1 and found that translational regulation of TOP transcripts was affected when mTORC1 signaling was disrupted [17]. The 4E-BP proteins downstream of mTORC1 were shown to play a role in the decrease of TOP protein synthesis when mTORC1 was inhibited [17]. Knockout of 4E-BP allowed TOP mRNAs to be ‘immune’ to repression by the mTORC1 inhibitor, Torin1. However, Miloslavski et al. later showed that 4E-BPs did not have a role in regulation of TOP transcript translation during physiological stresses such as oxygen deprivation and nutrient starvation [14]. Rather, Miloslavski suggests that the TOP transcript regulation during oxygen deprivation could be through an mTORC1-independent pathway and that the reliance on 4E-BP seen by Thoreen et al. can be attributed to a property of the cultured cell line used. 

More recent studies using compound screens have shown that TOP mRNA translation can also be regulated by mTORC1/4E-BP-independent pathways [17,18]. One such pathway was through the eIF2α kinase, GCN2. These findings point to a more complex regulatory circuitry for TOP transcripts. While early studies suggested that TOP translation can be regulated through mTORC1 or other nutrient sensing pathways [13,14], there has long been evidence of more direct means of regulation. In vitro competition studies using ‘TOP-like’ synthetic RNA oligos revealed the existence of a trans-acting factor that could regulate the translation of TOP transcripts. A great deal of effort has been put into the identification and functional characterization of proteins that bind to the 5’ TOP motif. This review focuses on the role of 5’ TOP motif-binding proteins and their roles in the control and regulation of 5’ TOP mRNA translation.

It should be noted that ‘TOP-like’ motifs consisting of oligopyrimidine tracts found internally within a 5’ untranslated region (UTR) have been reported to be regulated by mTORC1 in a manner similar to TOP transcripts [17]. However, early work on 5’ TOP motifs by Avni et al. showed that moving the TOP motif away from the 5’ terminus of the mRNA transcript decouples it from metabolic regulation [10]. Furthermore, as will be discussed later in the review, the binding of the major TOP regulatory protein requires the presence of a m^7^G cap that would be absent from 5’ UTR internal, TOP-like motifs. Thus, regulation of TOP-like motifs may be occurring through different mechanisms than those that control 5’ TOP motifs.

## 4. The La Family of Proteins

**Figure 2:**
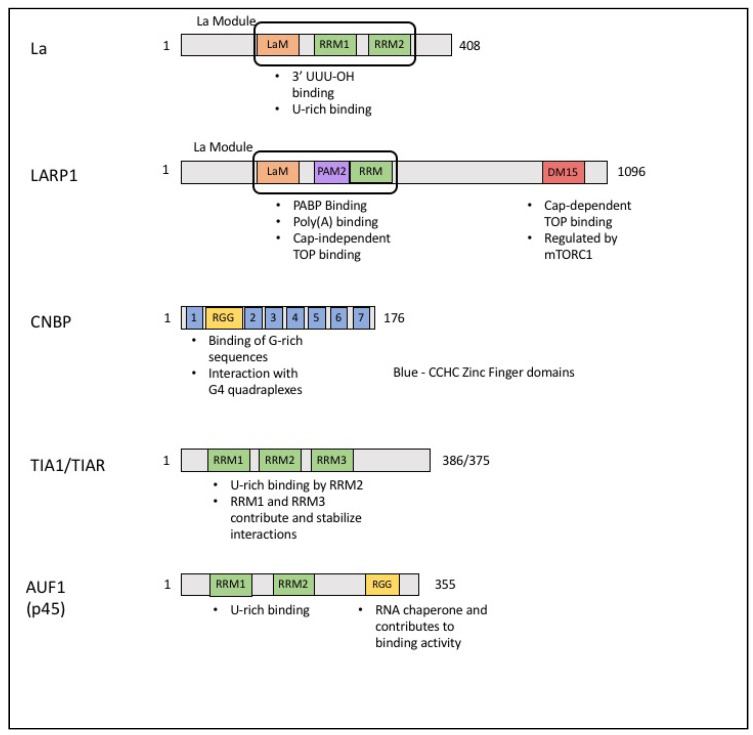
Schematics of 5’ TOP motif binding proteins and their RNA binding domains. La possesses the La Module consisting of the La Motif (LaM, orange) as well as two RNA recognition motifs (RRM, green). LARP1 also has the La module but with a poly(A) binding protein (PABP)-interacting motif (PAM2, purple). There is also the DM15 (red) domain in the C-terminal region that is required for 5’ TOP motif and cap binding. Cellular Nucleic Acid Binding Protein (CNBP) contains seven CCHC-zinc finger repeats (1-7, blue) as well as an arginine/glycine rich motif (RGG, yellow). AUF1 has four isoforms, p45 is the chosen canonical isoform shown here. AUF1 contains two RRMs as well as an RGG motif. All four isoforms contain the RRMs. In p42 and p45, the RGG is interrupted by an alternatively spliced exon.

One of the most widely studied families of RNA binding proteins (RBPs) is the La family [19]. La is a conserved RNA binding protein that is primarily nuclear. The ability of La proteins to bind to RNA is conferred by the La Module, a unique combination of a La motif (LaM) connected to an RNA Recognition Motif (RRM) [20,21,22,23] (Figure 2). The La Module is found in the La protein as well as in the La-related proteins (LARPs) [24,25]. RNA substrates for the different La family members can differ due to variations within the La Module [25]. La plays a key role in the biogenesis and maturation of RNA Polymerase III transcripts. During tRNA maturation, La binds to pre-mature tRNA through interactions with the 3’ UUU-OH feature found in RNA polymerase III transcripts [26,27,28]. Once bound to a pre-tRNA, La increases structural stability [29], protects from premature degradation [30], and acts as a chaperone during modification and splicing events [31,32].

La was first shown to associate with 5’ TOP mRNAs in *Xenopus laevis* [33]. Due to the affinity of La protein for 3’ UUU-OH motifs in associated transcripts, this interaction is likely mediated by interactions with oligo U tracts within the 5’ TOP motif. La was shown to bind to 5’ TOP motifs and stimulate translation in both *Xenopus laevis* and human cell lines [34]. In these studies, binding of La to TOP transcripts was found to be greater in growing *Xenopus laevis* cells than in resting cells. Interestingly, over-expression of La in resting cells led to an increase in TOP mRNA translation [34]. In further support of a stimulatory role, La was shown to associate with TOP transcripts during translation in vivo [35]. In contrast, other groups have reported that increasing amounts of non-phosphorylated La correlates with decreased translation of TOP mRNAs [36,37,38]. It has also been reported that La has no effect on TOP mRNA translation based on knockout and over-expression experiments [39]. Due to these conflicting results, the role of La in the translational regulation of TOP transcripts remains uncertain [11].

## 5. La Related Protein 1 (LARP1)

### 5.1. LARP1 Binds 5’ TOP Motifs in a Sequence Dependent Manner

While the La protein does not appear to play a direct role in 5’ TOP mRNA regulation, the closely related protein, La related protein 1 (LARP1), is a prime candidate for being a core regulator of TOP translation. Like La, LARP1 is a nuclear and cytosolic RNA-binding protein that contains a La module consisting of a LaM and RRM [40]. First identified as a protein that binds to TOP transcripts in *Xenopus laevis* [36,37], LARP1 binds to the 5’ TOP motif through its C-terminal DM15 domain [41]. Interestingly, binding of 5’ TOP motifs was shown to be independent of the La module [41]. The DM15 domain is a highly conserved combination of three HEAT-like helix-turn-helix regions. More complex studies using crystal structures of the human LARP1 DM15 region revealed that Lys-915 stabilizes the γ-phosphate of the m^7^G cap and the C1 nucleotide of the TOP motif interacts with Arg-879 and Arg-847 in the helix-turn-helix region [42]. See [42] for a detailed structural analysis of LARP1 and TOP motif interactions. These coordinating residues are conserved throughout worm, plants, and mammals. Using RNA electromobility shift assays (REMSA), Lahr et al. showed that binding of DM15 to RNA required both the m^7^G cap and the C1 nucleotide of the 5’ TOP motif. When the m^7^G cap was removed or C1 was mutated to G, DM15 binding affinity was decreased by approximately 90-fold [42]. This detail of DM15 binding poses an interesting challenge for studying LARP1. Commercially available RNA polymerases, using SP6 or T7 promoters, can only produce RNA transcripts beginning with guanine. To overcome this obstacle, several clever biochemical approaches have been employed. Production of short, chemically synthetized TOP motifs have been used to study binding dynamics of LARP1 [16,41,42]. A system utilizing cleavage by hammerhead ribozymes followed by splint ligation of a 5’ TOP oligo has also been used successfully to study the biological role a 5’ TOP motif has on translation [16,42].

### 5.2. LARP1 Represses Translation of TOP Transcripts

Early work in Drosophila identified LARP1 as a PABP binding partner that is an important player in cell proliferation and fertility [43]. It was also shown that depletion of LARP1 in the Human Embryonic Kidney cell line, HEK293 leads to a reduction in proliferation [16]. LARP1 has also been identified as a strongly expressed protein in several malignant cancers that correlates with progression and poor outcome [44,45,46,47]. The role of LARP1 on TOP RNA regulation was first reported based on its observed effect on RNA stability. Using RNA immunoprecipitation and proteomics, Aoki et al. identified LARP1 as a protein that binds to poly(A) tails of RNA transcripts [48]. They further showed that LARP1 was able to increase the steady state levels of mature TOP transcripts and concluded that LARP1 was a promoter of TOP protein expression [48]. To further support this, it has been shown that LARP1 can interact with the 40S ribosomal subunit and selectively stabilize TOP transcripts [49]. It has also been reported that LARP1 associates with translating ribosomes through interactions with PABP and is able to promote translation of mRNAs containing 5’ TOP motifs [40]. In contrast, Fonseca et al. reported LARP1 as a repressor for TOP translation that is regulated by mTORC1. In this study, LARP1 was able to compete with eIF4G binding to capped TOP transcripts and inhibit translation [13]. Fonseca suggests that the apparent discrepancy between these observations may be due a decrease in cell viability and protein synthesis when LARP1 is robustly knocked down [13]. Moderate LARP1 knockdown, as used in Fonseca’s study, resulted in less disruption of overall translation rates and allowed for the identification of an inhibitory effect. Several other groups have since reported the inhibitory effect of LARP1 on TOP translation [16,40,42].

### 5.3. LARP1 Binding and TOP Repression Is Controlled by mTORC1 Signaling

As TOP mRNA translation is strongly regulated by the mTORC1 pathway, it comes as no surprise that LARP1 is also a target of mTORC1 signaling. Using a proteomic approach, Fonseca et al. showed that LARP1 interacts with mTORC1 not only functionally, but also physically. It was further shown that this interaction was mediated by the regulatory-associated protein of mTOR (RAPTOR) [13]. This interaction with mTORC1 is important for LARP1’s ability to bind to TOP mRNAs. When mTORC1 was inactivated using rapamycin or Torin1, LARP1 interaction with TOP mRNAs was increased as measured by RNA immunoprecipitation. This increase in LARP1 binding was accompanied by a decrease of eIF4G interaction with TOP transcripts. Furthermore, when LARP1 was knocked down in HEK293T cells, inhibition of TOP translation by pharmacological agents was rescued. Phospho-proteomic studies have revealed several residues in LARP1 that are phosphorylated by mTORC1 [50,51,52]. Phosphorylation of two residues, Ser-744 and Ser-766, was shown to be inhibited by rapamycin and Torin1 [53]. Interestingly, these two residues are found near the DM15 region of LARP1 [41]. Philippe et al. showed that DM15 domain of LARP1 was necessary and sufficient for repression of TOP RNAs in both cells and in vitro systems, and also showed that this repression was responsive to mTOR inhibition by Torin1 [16]. Furthermore, it was shown that a region downstream of the DM15 domain allowed for regulation of LARP1 binding to TOP RNAs. This observation further adds evidence to potential mTORC1 phosphorylation sites regulating the interaction between LARP1 and 5’ TOP motifs. Intriguingly, it has also been reported that LARP1 stabilizes the mTOR mRNA likely through interactions with its 3’ UTR [54]. The possibility of a feedback loop between LARP1 and the mTOR pathway could lead to novel insights into the complexities of energy states and ribosome biogenesis.

### 5.4. LARP1 Inhibits Translation of TOP Transcripts by Competing with Initiation Factors

LARP1 has been shown to be a mTORC1 regulated protein that binds to 5’ TOP motifs and controls translation of TOP mRNAs. As previously discussed, canonical, cap-dependent translation requires binding of eIF4E to the cap structure with further recruitment of eIF4G and eIF4A to assemble the eIF4F complex that recruits the ribosome (Figure 1). Competition studies using REMSAs has shown that LARP1 competes with eIF4E for binding to the cap structure of TOP mRNAs [13]. More intriguingly, LARP1 is able to displace eIF4F from TOP RNAs even at sub-stoichiometric levels [42]. As demonstrated in other studies, LARP1 binding is highly dependent upon the nucleotide after the m^7^G cap being a cytosine, a pyrimidine, and binding is greatly diminished when it is replaced by a purine. Analysis of mRNA features for cap binding of eIF4E shows the opposite. eIF4E has a higher affinity for purines (A and G) over pyrimidines (C and U) [55,56]. This decreased affinity of eIF4E for 5’ TOP motifs could also explain why increasing 4E-BP expression appears to selectively inhibit TOP translation [17]. Based on this observation, it has been speculated that as 4E-BP levels increase and block the binding of eIF4E to cap structures, the transcripts with lower affinity for eIF4E binding, such as TOP transcripts, may be repressed earlier than transcripts with a higher affinity for eIF4E [17,57].

### 5.5. LARP1 Regulation of TOP Transcripts is Complex

**Figure 3:**
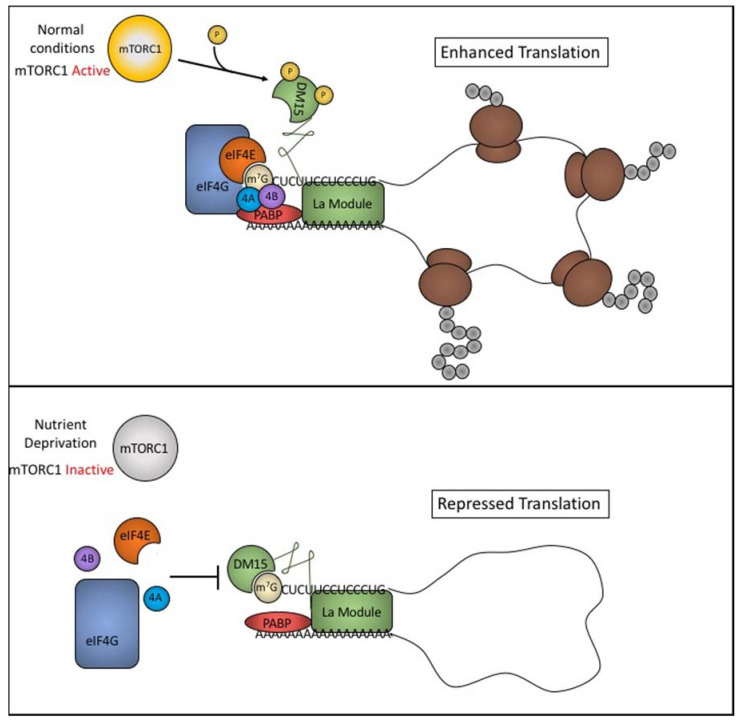
LARP1 regulates translation of 5’ TOP motif containing transcripts. Upper panel: Under normal nutrient conditions, the DM15 domain of LARP1 is unable to bind to the m7G cap allowing eIF4E binding, eIF4F assembly, and procession of translation initiation. The La module of LARP1 binds to the poly(A) tail, PABP, and the 5’ TOP motif in a cap independent manner. These interactions promote circularization of the TOP transcript and enhance translation. Lower panel: Nutrient deprivation leads to inactivation of mTORC1 and hypophosphorylation of residues in or near the DM15 domain allowing it to bind to the 5’ TOP motif and the m7G cap. DM15 blocks interactions of eIF4E with the cap and halts the downstream steps required for cap-dependent translation initiation thereby repressing translation.

As alluded to earlier, LARP1 also interacts with poly(A) tails of mRNA transcripts as well as with PABP [40,48,58]. This observation was further confirmed by Fonseca et al. and shown to be mediated by a PAM2-like domain found in the La module [13]. Deletion and mutation of this region resulted in a decrease in PABP binding by LARP1. It was further shown that the interaction with PABP was independent of RNA and mTORC1 regulation [13]. Recent work has revealed that LARP1 can also interact with the poly(A) tail through its La Module [58]. Here, Al-Ashtal et al. suggest a model in which LARP1 interacts with TOP transcripts in both nutrient rich and nutrient starved conditions through interactions with the La module. This observation may reconcile the apparent contradiction between descriptions of LARP1’s role in translation regulation. When mTORC1 is inactivated during nutrient deprivation, the DM15 domain of LARP1 is able to ‘clamp’ onto the 5’ cap of the TOP transcript. Fonseca et al. showed that inactivation of mTORC1 due to nutrient deprivation leads to an activation of LARP1 by hypophosphorylation [13]. The DM15 region of LARP1 then binds to the 5’ TOP motif and inhibits assembly of the eIF4G initiation complex thereby halting translation (Figure 3, upper panel). Together these data support a role for LARP1 in directly binding and controlling the fate of TOP mRNA translation. This regulation appears to be complex and can be tuned depending on the energy status of the cell.

## 6. Cellular Nucleic Acid Binding Protein (CNBP)

Cellular Nucleic Acid Binding Protein (CNBP) is a DNA and RNA binding protein that has been shown to bind to G-rich regions of 5’ TOP containing transcripts in both *Xenopus laevis* and humans [33,59,60]. Since its discovery, it has been implicated in the development of Myotonic dystrophy type 2 (DM2) [61]. DM2 is a multisystemic disease that is characterized by myotonia, muscle weakness, cardiac and endocrinological defects, and changes in cognitive functions [62]. Onset of DM2 is linked to an expansion of rCCTG repeats found in the first intron of CNBP [61]. Normally, this intron includes 11 to 26 CCTG repeats. In DM2 patients, this intron includes between 75 to 11,000 CCTG repeats [61]. These repeats lead to a reduction of CNBP expression that have been confirmed in animal models of DM2 [63] as well as muscle biopsies of DM2 patients [64]. Low levels of CNBP in DM2 patients were linked to a reduction in the expression of ribosomal proteins and other TOP transcript products including PABP and eEF2 [59]. A decrease in the transcripts that are required for protein synthesis leads to a decrease in overall protein production in patient-derived DM2 cell lines [59,65]. When CNBP was expressed in a DM2 myoblast model, TOP mRNA translation was restored [59,65]. These observations suggest that CNBP may have a positive role in TOP transcript translation.

Using PAR-CLIP analysis, Benhalevy et al. were able to identify G-rich CNBP binding sites in ~4000 mRNA transcripts, including TOP transcripts [66]. Many of these G-rich regions have also been shown to form G-quadruplex (G4) structures [67,68]. G4-structures are formed when tracts of guanines in an RNA or DNA interact through Hoogsteen base pairing [69]. The guanine repeats form a four-stranded structure that is highly stable. CNBP binds RNA via seven CCHC-Zinc Finger motifs and an arginine/glycine-rich motif (RGG motif). Benhalevy et al. used ribosome profiling to show that CNBP binding to G-rich regions increases translation. Depletion of CNBP increased the appearance of stalled ribosomes near predicted CNBP-binding sites [66]. Furthermore, CNBP was shown to inhibit the formation of G4 structures in a dose-dependent manner. These findings suggest that G4 structures inhibit ribosome elongation and that CNBP promotes translation by binding to and resolving G4 structures. 

While there is compelling evidence that CNBP binds downstream of 5’ TOP motifs and can potentially promote their translation, evidence that CNBP directly modulates the translation of TOP transcripts is lacking. In contrast, there is strong evidence that LARP1 represses the translation of TOP transcripts. It is nevertheless possible that both of these proteins contribute to this regulatory process. One attractive model is that LARP1 and CNBP bind to distinct regions of TOP transcripts and have opposing effects on translation (Figure 3). Futures studies clarifying the role of CNBP in the modulation of TOP transcript expression are needed.

## 7. T-Cell Intracellular Antigen (TIA1) and TIA-Related Protein (TIAR)

TIA1 and TIAR are closely related RBPs that share three RRMs and a prion-related carboxy-terminal domain. Sequence similarity between the three RRMs is high (79%, 89%, and 91%, respectively) and RRM2 has a high affinity for U-rich regions [70]. TIA1 and TIAR bind to Adenine/Uridine-rich elements (AREs) in the 3’ UTR of mRNA transcripts. AREs are commonly found in transcripts that encode proteins involved in proliferation, stress response, and immune functions [71,72]. When bound to these transcripts, TIA1 and TIAR function as translational repressors [73,74]. TIA1 and TIAR-dependent translational repression is enhanced in cells exposed to environmental stress. Phosphorylation of eIF2α during stress induces assembly of stalled 48S pre-initiation complexes (PIC). TIA1 and TIAR bind to PIC-bound transcripts whereupon their prion-related domains promote condensation into stress granules [75]. Protein and RNA content of stress granules is dynamic and selective based on several factors such as severity and nature of cellular stress and the expression of stress granule-associated proteins [76,77,78]. With the resolution of stress, stress granule disassembly correlates with the resumption of protein synthesis [79]. 

## 8. TIA1 and TIAR in TOP Regulation

TIA1 and TIAR also bind to 5’ TOP motifs as shown in experiments using Systematic Evolution of Ligands by Exponential enrichment (SELEX) analysis, gene array studies, and RNA immunoprecipitation [72,80,81]. This binding is likely due to the U-rich nature of the pyrimidine tracts. Damgaard and Lykke-Anderson showed that amino acid starvation triggers the recruitment of TIA1 and TIAR to 5’ TOP transcripts and inhibits translation. Interestingly, transcripts that have TIA1/TIAR binding sites within their 3’ UTR, but not a 5’ TOP motif, are exempt from regulation due to amino acid starvation [80]. This study also showed that TOP transcript repression by TIA1 and TIAR is dependent on GCN2 signaling. As mentioned above, stress granule formation depends on phosphorylation of eIF2α to deplete translationally competent initiation complexes. GCN2 activation induces phosphorylation of eIF2α and triggers stress granule formation. GCN2 activation and the selective binding of TIA/TIAR to 5’ TOP transcripts work together to repress production of TOP proteins by sequestering their transcripts into stress granules during amino acid starvation. Further studies to determine how TIA1 and TIAR interact with the 5’ TOP motif and translation initiation factors need to be performed to completely understand the mechanism of this observation.

## 9. ARE/poly(U)-Binding/Degradation Factor 1 (AUF1)

ARE/poly(U)-binding/degradation factor 1 (AUF1) is a family of proteins consisting of four isoforms generated by alternative splicing [82]. The four isomers have differing molecular weights (p37, p40, p42, p45) and form stable dimers in both the nucleus and cytoplasm, though not in equal proportions [82]. Each of these isoforms contains two conserved RRMs and an RGG motif (Figure 2). These motifs are necessary for AUF1 to recognize and bind to U-rich regions [83,84]. While the RRMs of AUF1 alone can bind RNA, high affinity interactions require the RRMs as well as the RGG motif found in the C-terminus of the protein [85]. The RNA affinity for each of the AUF1 isoforms are not identical due to the absence of different retained introns [86]. 

AUF1 functions in both mRNA turnover and translational control. AUF1 binds to AREs in the 3’ UTR of transcripts and is thought to recruit other protein factors that facilitate mRNA decay [87]. However, there are also reports of AUF1 binding and stabilizing other transcripts such as IL-6 and TGF-β in breast fibroblast cell lines [88,89]. More recently, AUF1 was shown to stabilize both VEGF-A and HIF1α by binding to sequences in their 3’ UTRs [90]. Interestingly, AUF1 has been shown to compete with TIAR for binding to *MYC* transcripts [91]. In this system, TIAR suppresses Myc expression, while binding of AUF1 in place of TIAR supports translation of Myc. However, the role of AUF1 in translational control is not well understood. Ribosome profiling experiments have revealed that AUF1 levels can affect ribosome occupancy on a number of transcripts [92]. The majority of these AUF1-associated transcripts do not undergo changes in steady state mRNA levels. These observations suggest that AUF1 is controlling translation by a mechanism other than mRNA stability or decay.

**Figure 4:**
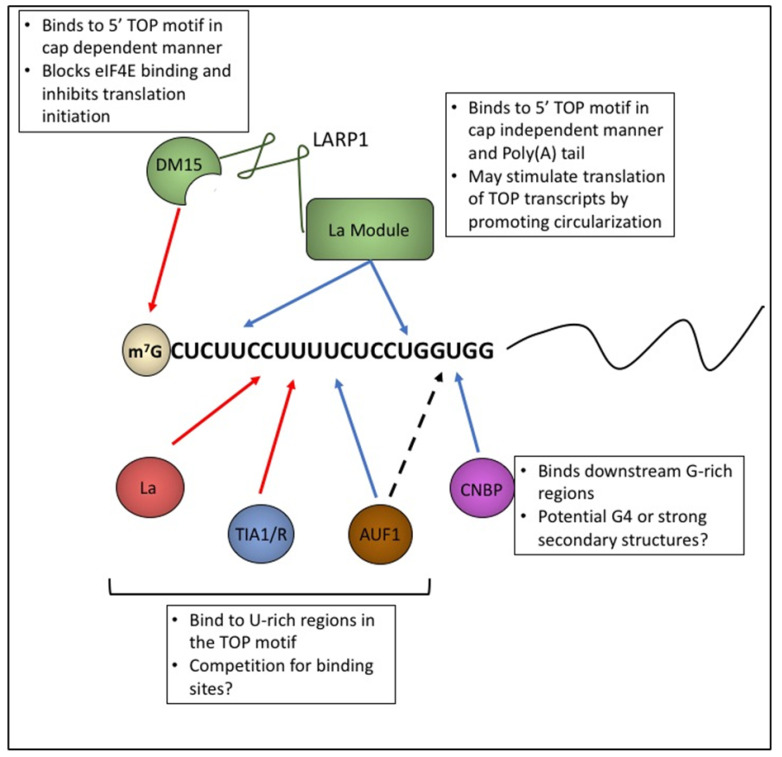
Multiple proteins bind to 5’ TOP motifs and affect translation. Schematic depicting potential interactions of RNPs with the 5’ TOP motif. Red arrows depict inhibition of expression and blue arrows depict promoting effects on expression. Different part of LARP1 (green) can interact with the TOP motif and can both promote or repress translation depending on the context. La (red) and TIA1/R (blue) bind to U-rich regions of the TOP motif and are thought to play an inhibitory role in TOP expression. AUF1 (brown) also binds to U-rich regions of the TOP motif but appears to have a promoting effect on expression. Furthermore, La, TIA1/R, and AUF1 may compete for binding sites. CNBP interacts with G-rich regions near the distal part of the TOP motif. CNBP as well as AUF1 may associate with secondary structures and potential G4 quadraplexes that may form in this region of some TOP motifs through their RGG motifs.

While AUF1 is primarily associated with transcript stability and translational control, it was also identified as a protein that binds to 5’ TOP motifs in an mTOR dependent manner [93]. Using modified tandem column chromatography, Kakegawa et al. were able to isolate and identify two protein that bound to the 5’ TOP motif of RPL32 in vitro. Further analysis identified these proteins as the p42 and p45 isoforms of AUF1. Levels of p42 and p45 AUF1 were decreased in polysome fractions when BJAB cells, a malignant human B-cell line, were treated with rapamycin to inhibit translation of TOP transcripts. This result suggests that AUF1 may have a stimulatory effect on TOP translation. Unfortunately, the role of AUF1 in TOP translation has not been widely studied. As such, direct evidence for AUF1 binding to TOP transcripts and regulating their expression has yet to be shown. As mentioned above, there is precedent for competition of AUF1 and TIA1/TIAR for common binding sites [91]. Competitive binding between these proteins may be a regulatory point for the translation of TOP transcripts (Figure 4).

## 10. Conclusions

Expression of ribosomal proteins and other factors required for protein synthesis is key for cellular function. Because protein synthesis is an energy intensive process, a gatekeeper that ensures that energy conditions are sufficient to initiate this program is essential. The 5’ TOP motif found in transcripts encoding all human ribosomal proteins, as well as a host of initiation factors, elongation factors, and other proteins required for translation is a good candidate for such a gatekeeper. In this review, the proteins that bind to 5’ TOP motifs and their activities have been discussed. While our understanding of the individual roles each of the binding proteins plays on TOP transcripts is growing, we are still uncovering the complexities of how these proteins are regulated and the downstream consequences of that regulation on protein synthesis. Furthermore, how these proteins interact with each other in the context of binding and regulating expression of TOP transcripts has yet to be elucidated. LARP1, CNBP, AUF1, and TIAR/1 have all been reported to be in stress granules [75,94,95,96]. Colocalization within stress granules may provide opportunity for these proteins to interact. Moving forward, studies highlighting the potential for interplay between different TOP binding proteins and their associated regulatory pathways will lead to a greater understanding of how protein synthesis and ribosome production are modulated during stress events in cells.

## Appendix A

**Table A1:** List of transcripts containing 5’ TOP motifs. The first 50 nucleotides of each TOP transcript. The 5’ TOP motif is highlighted in yellow and guanines are in red.

Gene	5’ UTR (First 50 Nucleotides)	Accession No.
*RPSA*	CUUUUUCCGUGCUACCUGCAGAGGGGUCCAUACGGCGUUGUUCUGGAUUC	D28372.1
*RPS2*	CUUCUUUUCCGACAAAACACCAAAUGGCG	AB055772.1
*RPS3*	CCUUUCCUUUCAGCGGAGCGCGGCGGCAAGAUGGCA	D28344.1
*RPS3A*	CCUUUUGGCUCUCUGACCAGCACCAUGGCG	D28374.1
*RPS4X*	CUCUUUCCUUGCCUAACGCAGCCAUGGCU	D28359.1
*RPS4Y*	CUCUUCCGUCGCAGAGUUUCGCCAUGGCC	AB055773.1
*RPS5*	CUCUUCCUGUCUGUACCAGGGCGGCGCGUGGUCUACGCCGAGUGACAGAG	D28455.1
*RPS6*	CUCUUUUCCGUGGCGCCUCGGAGGCGUUCAGCUGCUUCAAGAUGAAG	D28348.1
*RPS7*	CUCUUCCUAAGCCGGCGCUCGGCAAGUUCUCCCAGGAGAAAGCCAUGUUC	AB055774.1
*RPS8*	CUCUUUCCAGCCAGCGCCGAGCGAUGGGC	D28361.1
*RPS9*	CUCUUUCUCAGUGACCGGGUGGUUUGCUUAGGUGAGGUGCGG	AB061839.1
*RPS10*	CUUUUCCAGCCCCGGUACCGGACCCUGCAGCCGCAGAGAUGUUG	D28427.1
*RPS11*	CUUUUUUUUUUUCAGGCGGCCGGGAAGAUGGCG	D28407.1
*RPS12*	CUUUCCCUGCCGCCGCCGAGUCGCGCGGAGGCGGAGGCUUGGGUGCGUUC	D28378.1
*RPS13*	CCUUUCGUUGCCUGAUCGCCGCCAUCAUGGGU	D28429.1
*RPS14*	CUUUUUCCGGUGUGGAGUCUGGAGACGACGUGCAGAAAUGGCA	D28352.1
*RPS15*	CUUCUGAGGAUCCGGCAAGAUGGCA	AB055776.1
*RPS15A*	CUCUUUCCGCCAUCUUUCCGCGCCGCCACAAUGGUG	D28347.1
*RPS16*	CUUUUCCGGUUGCGGCGCCGCGCGGUGAGGUUGUCUAGUCCACGCUCGGA	D28392.1
*RPS17*	CUCUUUUACCAAGGACCCGCCAACAUGGGC	AB055777.1
*RPS18*	CUCUUCCACAGGAGGCCUACACGCCGCCGCUUGUGCUGCAGCCAUGUCU	AB055778.1
*RPS19*	CUUUCCCCUGGCUGGCAGCGCGGAGGCCGCACGAUGCCU	D28389.1
*RPS20*	CUUUUUUUUUGAGGAAGACGCGGUCGUAAGGGCUGAGGAUUUUUGGUCCG	D28358.1
*RPS21*	CUUUUCUCUCUCGCGCGCGGUGUGGUGGCAGCAGGCGCAGCCAGCCUCGA	D28422.1
*RPS23*	CUCUUUCGCUCAGGCCCGUGGCGCCGACAGGAUGGGC	D28396.1
*RPS24*	CUCUUUUCCUCCUUGGCUGUCUGAAGAUAGAUCGCCAUCAUGAAC	D28424.1
*RPS25*	CUUCCUUUUUGUCCGACAUCUUGACGAGGCUGCGGUGUCUGCUGCUAUUC	D28369.1
*RPS26*	CUCCUCUCUCCGGUCCGUGCCUCCAAGAUGACA	AB056456.1
*RPS27*	CUUUCCGGCGGUGACGACCUACGCACACGAGAACAUGCCU	D28454.1
*RPS27A*	CUUCCUUUUCGAUCCGCCAUCUGCGGUGGAGCCGCCACCAAAAUGCAG	D28404.1
*RPS28*	CUCUCCGCCAGACCGCCGCCGCGCCGCCAUCAUGGAC	AB055779.1
*RPS29*	CCUUUUACCUCGUUGCACUGCUGAGAGCAAGAUGGGU	AB055780.1
*RPS30*	CUCUUUCUCGACUCCAUCUUCGCGGUAGCUGGGACCGCCGUUCAGUCGCC	D28403.1
*RPP0*	CUCUCGCCAGGCGUCCUCGUGGAAGUGACAUCGUCUUUAAACCCUGCGUG	D28418.1
*RPP1*	CUUUUUUUCCUCAGCUGCCGCCAAGGUGCUCGGUCCUUCCGAGGAAGCUA	D28366.1
*RPP2*	CCUUUUCCUCCCUGUCGCCACCGAGGUCGCACGCGUGAGACUUCUCCGCC	D28411.1
*RPL3*	CUCUACCGGCGGGAUUUGAUGGCGUGAUGUCU	D28415.1
*RPL4*	CUUUUCCUGUGGCAGCAGCCGGGCUGAGAGGAGCGUGGCUGUCUCCUCUC	D23660.1
*RPL5*	CUUUUCCCACCCCCUAGCGCCGCUGGGCCUGCAGGUCUCUGUCGAGCAGC	AB055762.1
*RPL6*	CUUUCUUUCCCAUCUUGCAAGAUGGCG	D28388.1
*RPL7*	CUCUUUUUCCGGCUGGAACCAUGGAG	AB055763.1
*RPL7A*	CUCUCUCCUCCCGCCGCCCAAGAUGCCG	D28405.1
*RPL8*	CUCUUUCGGCCGCGCUGGUGAACAGGACCCGUCGCCAUGGGC	D28421.1
*RPL9*	CUUUUUUUGCUGCGUCUACUGCGAGAAUGAAG	D28399.1
*RPL10*	CUCUUUCCCUUCGGUGUGCCACUGAAGAUCCUGGUGUCGCCAUGGGC	D28410.1
*RPL11*	CUCUUCCUGCUCUCCAUC	NM_000975.5
*RPL10A*	CUCUUUUCCGGUUAGCGCGGCGUGAGAAGCCAUGAGC	AB055764.1
*RPL12*	CUCUCGGCUUUCGGCUCGGAGGAGGCCAAGGUGCAACUUCCUUCGGUCGU	D28443.1
*RPL13*	CUUUCCGCUCGGCUGUUUUCCUGCGCAGGAGCCGCAGGGCCGUAGGCAGC	NM_000977.4
*RPL13A*	CUUUUCCAAGCGGCUGCCGAAGAUGGCG	D28409.1
*RPL14*	CUUCUUCCUUCUCGCCUAACGCUGCCAACAUGGUG	AB055765.1
*RPL15*	CCUUUCCGUCUGGCGGCAGCAUCAGGUAAGCCAAGAUGGGU	D28417.1
*RPL17*	CUCUUUCCCUAAGCAGCCUGAGGUGAUCUGUGAAAAUGGUU	D28373.1
*RPL18*	CUUUCCGGACCUGGCCGAGCAGGAGGCGCAAUCAUGGGA	D28461.1
*RPL18A*	CUUUUGCGGGUGGCGGCGAACGCGGAGAGCACGCCAUGAAG	D28393.1
*RPL19*	CUUUCCUUUCGCUGCUGCGGCCGCAGCCAUGAGU	AB055766.1
*RPL21*	CCUUUCGGCCGGAACCGCCAUCUUCCAGUAAUUCGCCAAA	D28406.1
*RPL22*	CUUUUUUUUUCUAACUCCGCUGCCGCC	D28346.1
*RPL23*	CCUUUUUUCUUUUUUCCGGCGUUCAAG	D28349.1
*RPL23A*	CCUUUUCACAAG	D28401.1
*RPL24*	CUUUUCUUUCGCCAUCUUUUGUCUUUCCGUGGAGCUGUCGCC	D28400.1
*RPL26*	CUCUUCCCUUUUGCGGCCAUCACCGAAGCGGGAGCGCCAAA	D28413.1
*RPL27*	CCUUUUUGCUGUAGGCCCGGGUGGUUGCUGCCGAA	D28453.1
*RPL27A*	CUUUUUCGUCUGGGCUGCCAAC	AB055767.1
*RPL30*	CUUUUCUCGUUCCCCGGCCAUCUUAGCGGCUGCUGUUGGUUGGGGGCCGU	D28438.1
*RPL31*	CUUCCUUUCCAACUUAGACGCUGCAGA	D28386.1
*RPL32*	CUUCCUCGGCGCUGCCUACGGAGGUGGCAGCCAUCUCCUUCUCGGCAUCA	D28385.1
*RPL34*	CUUCCUCUUCCGGGGACGUUGUCUGCAGGCACUCAGAAUGGUC	D28420.1
*RPL35*	CUCUUUCCCUCGGAGCGGGCGGCGGCGUUGGCGGCUUGUGCAGCAAUGGC	D28448.1
*RPL36*	CUUUGCGCCACGGCCGUCUCUGGAGAGCAGCAGCCAUGGCC	AB055769.1
*RPL36A*	CUUUUUCCGCGCCGAUAGCGCUCACGCAAGCAUGGUU	D28414.1
*RPL37*	CUUUCUGGUCUCGGCCGCAGAAGCGAGAUGACG	AB055770.1
*RPL39*	CUUCCGCCAGCUUCCCUCCUCUUCCUUUCUCCGCCAUCGUGGUGUGUUCU	D28397.1
*RPL40*	CUUCUUUUUCUUCAGCGAGGCGGCCGAGCUGGUUGGUGGCGGCGGUCGUG	NM_001321022
*RPL41*	CUCUCGGCCUUAGCGCCAUUUUUUUGGAAACCUCUGCGCCAUGAGA	D28462.1
*eIF3A*	CUCCUUCCUUUCCGUCUCUGGCCGGCUGGGCGCGGGCGACUGCUGGCGAG	JX312508.1
*eIF3E*	CUUUUCUUUGGCAAGAUGGCGGAGUACGACUUGACUACUCGCAUCGCGCA	NM_001568.3
*eiF3F*	CUUCUUUCUCGACAAGAUGG	NM_003754.3
*eIF3H*	CUCUUUCUUCCUGUCUGCUUGGAAAGAUG	NM_003756.3
*eIF4B*	CUUUUGCGUUCUCUUUCCCUCUCCCAACAUG	NM_001300821
*eEF1A*	CUUUUUCGCAACGGGUUUGCCGCCAGAACACAGGUGUCGUGAAAACUACC	NM_001402.6
*eEF1B2*	CCUUUUUCCUCUCUUCAGCGUGGGGCGCCCACAAUUUGCGCGCUCUCUUU	NM_001959.4
*eEF1D*	CUCCCUUUCAUCAGUCUUCCCGCGUCCGCCGAUUCCUCCUCCUUGGUCGC	NM_032378.6
*eEF1G*	CCUUUCUUUGCGGAAUCACCAUG	NM_001404.5
*eEF2*	CUCUUCCGCCGUCGUCGCCGCCAUCCUCGGCGCGACUCGCUUCUUUCGGU	NM_001961.4
*Rack1*	CUCUCUUUCACUGCAAGGCGGCGGCAGGAGAGGUUGUGGUGCUAGUUUCU	NM_006098.5
*PABP*	CCCCUUCUCCCCGGCGGUUAGUGCUGAGAGUGCGGAGUGUGUGCUCCGGG	NM_002568.4
*hnRNP A1*	CCUUUCUGCCCGUGGACGCCGCCGAAGAAGCAUCGUUAAAGUCUCUCUU	NM_002136.4
*Nucleophosmin*	CUUUCCCUGGUGUGAUUCCGUCCUGCGCGGUUGUUCUCUGGAGCAGCGUU	NM_002520.6
*NAP1L1*	CUUUUUUAGCGCCAUCUGCUCGCGGCGCCGCCUCCUGCUCCUCCCGCUGC	NM_139207.5
*TCTP*	CUUUUCCGCCCGCUCCCCCCUCCCCCCGAGCGCCGCUCC	NM_00128627
*Vimentin*	CCUCUGCCACUCUCGCUCCGAGGUCCCCGCGCCAGAGACGCAGCCGCGCU	NM_003380.5
